# Four novel *Pleurocordyceps* (Polycephalomycetaceae) species from China

**DOI:** 10.3389/fmicb.2023.1256967

**Published:** 2024-01-10

**Authors:** Yuan-Pin Xiao, Yu Yang, Ruvishika S. Jayawardena, Eleni Gentekaki, Xing-Can Peng, Zong-Long Luo, Yong-Zhong Lu

**Affiliations:** ^1^School of Food and Pharmaceutical Engineering, Guizhou Institute of Technology, Guiyang, China; ^2^Center of Excellence in Fungal Research, Mae Fah Luang University, Chiang Rai, Thailand; ^3^School of Science, Mae Fah Luang University, Chiang Rai, Thailand; ^4^University of Nicosia School of Veterinary Medicine, Nicosia, Cyprus; ^5^Engineering and Research Center for Southwest Bio-Pharmaceutical Resources of National Education Ministry of China, Guizhou University, Guiyang, China; ^6^College of Agriculture and Biological Sciences, Dali University, Dali, China

**Keywords:** entomopathogenic fungi, morphology, Polycephalomycetaceae, phylogeny, taxonomy

## Abstract

Entomopathogenic fungi comprise an ecologically important group of specialized pathogens infecting other fungi, invertebrates, and plants. These fungi are species-rich with high diversity and broad distribution worldwide. The majority of entomopathogenic fungi belong to clavicipitoids, which consist of the hypocrealean families, Clavicipitaceae, Cordycipitaceae, Ophiocordycipitaceae, and Polycephalomycetaceae. The latter is a newly established entomopathogenic family that recently separated from the family Ophiocordycipitaceae to accommodate the genera, *Perennicordyceps, Pleurocordyceps*, and *Polycephalomyces*. In recent years, Polycephalomycetaceae has been enriched with parasitic and hyperparasitic fungi. With 16 species spread across China, Ecuador, Japan, and Thailand, *Pleurocordyceps* is the most speciose genus in the family. In this study, we expand the number of taxa in the genus by introducing four new *Pleurocordyceps* species from China, namely, *P. clavisynnema, P. multisynnema, P. neoagarica*, and *P. sanduensis*. We provide detailed descriptions and illustrations and infer genus-level phylogenies based on a combined 6-loci gene sequence dataset comprising the internal transcribed spacer gene region (ITS), small subunit ribosomal RNA gene region (SSU), large subunit rRNA gene region (LSU), translation elongation factor 1-alpha gene region (TEF-1α), RNA polymerase II largest subunit gene region (RPB1), and RNA polymerase II second largest subunit (RPB2). This study contributes to knowledge with regard to the diversity of *Pleurocordyceps* specifically and entomopathogenic *Hypocreales* more broadly.

## Introduction

Insect pathogenic fungi, also known as entomopathogenic fungi, comprise a group of over 2,000 species spanning 90 genera (Saltamachia and Araujo, 2020). The phylogenetic diversity of entomopathogenic fungi is notable, with the majority belonging to *Hypocreales*, the largest group of plant and insect pathogens in *Sordariomycetes* (Sung et al., 2007; Maharachchikumbura et al., 2016; Wijayawardene et al., 2018). Within *Hypocreales*, the families Clavicipitaceae, Cordycipitaceae, Ophiocordycipitaceae, and Polycephalomycetaceae are collectively known as the clavicipitoid fungi and contain the majority of known insect pathogens (Hyde et al., 2020; Wei et al., 2020; Wijayawardene et al., 2020; Huang et al., 2021; Xiao et al., 2023). Some species are well known in the fields of agriculture and related industries, including *Beauveria bassiana* (biological control agent), *Cordyceps militaris* (medicinal), *Metarhizium anisopliae* (biological control agent), and *Ophiocordyceps sinensis* (medicinal) (Zimmermann, 2007; Acuña Jiménez et al., 2015; Li et al., 2020; Eiamthaworn et al., 2022). Thus, entomopathogenic fungi have gained the attention of researchers as a crucial fungal resource (Fernández-Grandon et al., 2020; Sharma et al., 2020; Sobczak et al., 2020; Zha et al., 2021).

The taxonomy of entomopathogenic fungi has undergone substantial changes since the advent of the molecular era (Tasanathai et al., 2016; Dong et al., 2022). Chaverri et al. (2005) initiated this molecular exploration by providing LSU, TEF, and RPB1 data for *Polycephalomyces formosus* and *Polycephalomyces ramosopulvinatus* (current name: *Pleurocordyceps ramosopulvinata*). Ban et al. (2009) used a 504-base-pair LSU fragment, but it fell short in resolving deep fungal nodes (Kepler et al., 2013). Different loci were selected for the analysis of novel species, with Wang et al. (2014) using a 4-loci (SSU, LSU, TEF, and RPB1), Wang et al. (2015b) using a 5-loci (SSU, LSU, TEF, RPB1, and RPB2), and Wang et al. (2015a) and Xiao et al. (2018) utilizing a 6-loci (ITS, SSU, LSU, TEF, and RPB1, and RPB2). The phylogenetic placement of *Polycephalomyces* or the segregation of new genera from *Polycephalomyces* was analyzed using both 5-loci (SSU, LSU, TEF, RPB1, and RPB2) and 6-loci (ITS, SSU, LSU, TEF, RPB1, and RPB2) (Kepler et al., 2013; Matočec et al., 2014; Wang et al., 2021). Building on this molecular groundwork, Xiao et al. (2023) established a new family, Polycephalomycetaceae, accommodating three genera (*Perennicordyceps, Pleurocordyceps*, and *Polycephalomyces*) and comprising 28 species using 6 loci (ITS, SSU, LSU, TEF, RPB1, and RPB2).

Over the past decade, a multitude of new species have been described in the family Polycephalomycetaceae, including those documented by Kepler et al. (2012), Wang et al. (2015a,b), and Yang et al. (2020), contributing to a deeper understanding of its classification. Recent studies by Wei et al. (2022) and Xiao et al. (2023) have introduced additional new species, sparking renewed interest in the taxonomy of the family. The sexual morph of Polycephalomycetaceae is distinguished by producing superficial or immersed ascomata with a stipe, three layers of peridium, narrowly cylindrical asci, multiseptate ascospores, and short cylindrical part spores (Matočec et al., 2014; Wang et al., 2021; Xiao et al., 2023). Its asexual morphs have congregated mycelia on the surface of the host, light-colored synnemata with stipules, divergent conidiophores, and one or both types of phialides and conidia (Matočec et al., 2014; Wang et al., 2021; Xiao et al., 2023). Most species in Polycephalomycetaceae are found in tropical and subtropical regions, with fewer taxa found in temperate regions (Van Vooren and Audibert, 2005; Wang et al., 2012, 2015a; Matočec et al., 2014; Xiao et al., 2018, 2023). A high diversity of polycephalomycetous fungi has been found in China and Japan (Kobayasi, 1939, 1941; Kobayasi and Shimizu, 1982; Chen et al., 1984; Wang et al., 2012, 2014, 2015a,b, 2021; Kepler et al., 2013; Quandt et al., 2014; Yang et al., 2020; Xiao et al., 2023).

With 16 species, *Pleurocordyceps* is the most speciose genus in the family Polycephalomycetaceae (Wang et al., 2021; Xiao et al., 2023). *Pleurocordyceps* was established by Wang et al. (2021) with the type species, *P. sinensis*, which was found on *Ophiocordyceps sinensis* (Chen et al., 1984). *Pleurocordyceps* is distinguished from closely related genera by its lateral fertile pulvinate stromata near the tip of the sexual morph and its two types of phialides and conidia in the asexual morph (Wang et al., 2021; Xiao et al., 2023). Wang et al. (2021) provided a key to the 10 accepted *Pleurocordyceps* species (Wang et al., 2021; Xiao et al., 2023). The insect host orders associated with *Pleurocordyceps* sp. comprise *Coleoptera, Hymenoptera, Hemiptera, Lepidoptera, Orthoptera*, and *Homoptera* (Kobayasi, 1939; Kobayasi and Shimizu, 1982; Bischoff et al., 2003; Ban et al., 2009; Wang et al., 2012, 2015a,b; Crous et al., 2017; Xiao et al., 2018; Poinar and Vega, 2020). In addition to parasitizing insects, most species in the genus are also parasites of fungi (Kobayasi, 1941; Seifert, 1985; Bischoff et al., 2003; Ban et al., 2009; Wang et al., 2015a; Xiao et al., 2023). In recent years, *Ophiocordyceps* sp. has been frequently reported as the host of *Polycephalomyces*-like species (Sun et al., 2019; Xiao et al., 2023). Specifically, *Pleurocordyceps agarica, P. aurantiacus, P. lianzhouensis, P. sinensis*, and *P. yunnanensis* are parasites on *Ophiocordyceps* sp. and insects (Chen et al., 1984; Wang et al., 2012, 2015a,b, 2021; Xiao et al., 2018). In general, *Pleurocordyceps* spp. exhibit significant potential for producing a diverse range of secondary metabolites. For instance, *Pleurocordyceps nipponicus* and *P. phaothaiensis* contain natural antioxidant, antibacterial, antitumorigenic, anti-inflammatory, and antimicrobial compounds (Sangdee et al., 2017; Somsila et al., 2018; Sonyot et al., 2020). Gokhale et al. (2020) reported that the secondary metabolites of *P. sinensis* have antibacterial potential. However, there are noticeable gaps in critical areas, such as chemistry, industry, and ecology of *Pleurocordyceps* species. Thus, there is a compelling need for further research to explore the wide array of capabilities and applications within *Pleurocordyceps*.

In China, there are records of nine *Pleurocordyceps* species, along with more than 200 taxa of clavicipitoid fungi that have been found in the country (Wang et al., 2012, 2014, 2015a,b; Liang et al., 2016; Yang et al., 2020; Xiao et al., 2023). In this study, we introduce four new species of *Pleurocordyceps*, namely, *P. clavisynnema, P. multisynnema, P. neoagarica*, and *P. sanduensis*. We provide a detailed morphological description along with phylogenetic analyses using a combined 6-loci gene region (ITS, SSU, LSU, *tef-1*α, *rpb1*, and *rpb2*).

## Materials and methods

### Sample collection, isolation, and morphological studies

Fresh specimens, comprising a total of eight, were collected from soil in Anhui and Guizhou provinces, China. The samples were transported in plastic boxes to the laboratory, and pertinent metadata (location, longitude, and latitude) were recorded. The fruiting bodies were examined using a stereomicroscope (SMZ 745 and SMZ 800N, Nikon, Tokyo, Japan) and free-hand sections were obtained for analysis. Micromorphological features such as synnemata, conidiophores, phialides, and conidia were captured using a Nikon DS-Ri2 digital camera connected to a Nikon ECLIPSE microscope (Tokyo, Japan). The strains were obtained from fresh tissue by removing a small piece of mycelium from the host, which was then transferred with a sterile needle onto PDA plates and incubated at 25°C. The pure culture was stored in the Guizhou Culture Collection, China (GZCC). The specimens were deposited at the Guizhou Institute of Technology Herbarium (Herb. GZLG). The guidelines of the Facesoffungi database (https://www.indexfungorum.org) were followed to obtain Index Fungorum numbers, as outlined by Jayasiri et al. (2015). The morphological structures were measured using Tarosoft (R) v.0.9.7 Image Frame Work, and the photographic plates were processed using Adobe Photoshop CC 2022 (Adobe Systems, USA).

### DNA extraction, PCR amplification, and sequencing

Total DNA was extracted from fruiting bodies and cultures using the Fungal DNA MiniKit (Biotech, USA), following the manufacturer's instructions. Internal transcribed spacer gene region (ITS), small subunit ribosomal RNA gene region (SSU), large subunit rRNA gene region (LSU), RNA polymerase II largest subunit gene region (*rpb1*), RNA polymerase II second largest subunit (*rpb2*), and translation elongation factor 1-alpha gene region (*tef-1*α) gene amplifications were performed using the ITS5/ITS4, NS1/NS4, LR0R/LR5, CRPB1A/RPB1Cr, fRPB2-5F/fRPB2-7Cr, and 983F/2218R primers, respectively (Vilgalys and Hester, 1990; White et al., 1990; Hopple and Vilgalys, 1999; Castlebury et al., 2004; Sung et al., 2007). Previous studies have demonstrated that the use of these six genetic loci optimally resolves the phylogenetic placement of the species *Pleurocordyceps* (Xiao et al., 2018, 2023; Wang et al., 2021; Wei et al., 2022). The nuclear gene amplification reactions followed the protocol outlined by Yang et al. (2021). PCR products were sent to Tsingke Biotechnology for sequencing (Chongqing, China). All newly generated sequences were uploaded to GenBank, and accession numbers were assigned (Table 1).

**Table 1:** Accession numbers of DNA sequences used in the phylogenetic analyses.

**Taxon**	**Strain**	**GenBank accessions**						**References**
		**ITS**	**SSU**	**LSU**	***rpb1***	***rpb2***	***tef-1**α*	
*Pleurocordyceps agarica*	YHHPA1305	KP276651	KP276655	-	KP276663	KP276667	KP276659	Wang et al., 2015b
*P. agarica*	YHCPA1307	KP276654	KP276658	-	KP276666	KP276670	KP276662	Wang et al., 2015b
*P. aurantiacus*	MFLUCC 17-2113	MG136916	MG136904	MG136910	MG136866	MG136870	MG136875	Xiao et al., 2018
*P. aurantiacus*	MFLU 17-1394	MG136918	MG136906	MG136912	MG136867	MG136872	MG136876	Xiao et al., 2018
***P. clavisynnema***	**GZLG 23-102**	**OQ968788**	**-**	**OQ968796**	**-**	**-**	**OQ982009**	**This study**
***P. clavisynnema***	**GZCC 22-2042**	**OQ968789**	**OQ968805**	**OQ968797**	**OQ981998**	**OQ982004**	**OQ982008**	**This study**
*P. formosus*	ARSEF1424	KF049661	KF049615	KF049634	KF049651	KF049671	KF049689	Kepler et al., 2013
*P. formosus*	MFLU 18-0162	MK863250	MK863043	MK863050	MK860188	-	-	Unpublished
*P. heilongtanensis*	KUMCC 3008	OQ172091	OQ172111	OQ172063	OQ459759	OQ459805	OQ459731	Xiao et al., 2023
*P. kanzashianus*	-	AB027371	AB027371	AB027325	-	-	-	Nikoh and Fukatsu, 2000
*P. lanceolatus*	GACPCC 17-2005	OQ172077	OQ172109	OQ172047	OQ459755	OQ459801	OQ459727	Xiao et al., 2023
*P. lanceolatus*	GACP 17-2004	OQ172076	OQ172110	OQ172046	OQ459754	OQ459800	OQ459726	Xiao et al., 2023
*P. lianzhouensis*	HIMGD20918	EU149921	KF226245	KF226246	KF226247	-	KF226248	Zhang et al., 2007
*P. lianzhouensis*	GIMYY9603	EU149922	KF226249	KF226250	KF226251	-	KF226252	Zhang et al., 2007
*P. marginaliradians*	MFLU 17-1582	MG136920	MG136908	MG136914	MG136869	MG271931	MG136878	Xiao et al., 2018
***P. multisynnema***	**GZLG 23-101**	**OQ968792**	**OQ968802**	**OQ968800**	**-**	**OQ982002**	**-**	**This study**
***P. multisynnema***	**GZCC 22-2041**	**OQ968793**	**OQ968803**	**OQ968801**	**OQ981997**	**OQ982003**	**OQ982010**	**This study**
***P. neoagarica***	**GZLG 23-103**	**OQ968790**	**-**	**OQ968795**	**-**	**-**	**-**	**This study**
***P. neoagarica***	**GZCC 22-2043**	**OQ968791**	**OQ968804**	**OQ968794**	**OQ981996**	**OQ981999**	**OQ982007**	**This study**
*P. nipponicus*	NHJ 4268	KF049657	KF049621	KF049639	MF416676	KF049676	MF416517	Kepler et al., 2013
*P. nipponicus*	BCC 1682	KF049664	KF049620	KF049638	-	-	KF049694	Kepler et al., 2013
*P. nipponicus*	NBRC 101408	JN943303	JN941751	JN941390	JN992485	-	-	Schoch et al., 2012
*P. nipponicus*	BCC 2325	KF049665	KF049622	KF049640	KF049655	KF049677	KF049696	Kepler et al., 2013
*P. nutansis*	GACP 19-1906	OQ172079	OQ172117	OQ172049	OQ459763	OQ459809	OQ459737	Xiao et al., 2023
*P. nutansis*	MFLU 21-0275	OQ172073	OQ172119	OQ172048	OQ459765	OQ459811	OQ459739	Xiao et al., 2023
*P. onorei*	BRA CR23902	KU898841	-	-	-	-	-	Crous et al., 2017
*P. onorei*	BRA CR23904	KU898843	-	-	-	-	-	Crous et al., 2017
*P. phaothaiensis*	BCC84557	MF959734	-	MF959738	MF959746	-	MF959741	Crous et al., 2017
*P. phaothaiensis*	BCC84553	MF959733	-	MF959737	MF959745	-	MF959742	Crous et al., 2017
*P. ramosus*	RUTPP	-	-	AY259543	-	-	-	Bischoff et al., 2003
*P. ramosus like*	NBRC 101760	MN586827	MN586818	MN586836	MN598042	MN598060	MN598051	Wang et al., 2021
*P. ramosus like*	NBRC 109984	MN586828	MN586819	MN586837	MN598043	-	MN598052	Wang et al., 2021
*P. ramosus like*	NBRC 109985	MN586829	MN586820	MN586838	MN598044	-	MN598053	Wang et al., 2021
*P. ramosopulvinatus*	EFCC 5566	KF049658	-	KF049627	KF049645	-	KF049682	Kepler et al., 2013
*P. ramosopulvinatus*	SU 65	-	-	DQ118742	DQ127244	-	DQ118753	Chaverri et al., 2005
*P. ramosopulvinatus*	-	AB027372	AB027326	-	-	-	-	Nikoh and Fukatsu, 2000
*P. sinensis*	CGMCC 3.19069	MH459160	MH454346	-	-	-	-	Sun et al., 2019
*P. sinensis*	CN 80 2	HQ832884	HQ832887	HQ832886	HQ832888	HQ832889	HQ832890	Wang et al., 2012
*P. sinensis*	HMAS 43720	NR 119928	-	NG 042573	-	-	KF049697	Wang et al., 2012
***P. sanduensis***	**GZLG 23-104**	**OQ968786**	**-**	**OQ968798**	**-**	**OQ982000**	**OQ982005**	**This study**
***P. sanduensis***	**GZCC 22-2044**	**OQ968787**	**OQ968806**	**OQ968799**	**OQ981995**	**OQ982001**	**OQ982006**	**This study**
*P. tomentosus*	BL4	KF049666	KF049623	KF049641	KF049656	KF049678	KF049697	Kepler et al., 2013
*P. vitellina*	KUMCC 3005	OQ172088	-	OQ172060	OQ459756	OQ459802	OQ459728	Xiao et al., 2023
*P. vitellina*	KUMCC 3006	OQ172089	-	OQ172061	OQ459757	OQ459803	OQ459729	Xiao et al., 2023
*P. vitellina*	KUMCC 3007	OQ172090	-	OQ172062	OQ459758	OQ459804	OQ459730	Xiao et al., 2023
*P. yunnanensis*	YHCPY1005	KF977848	KF977848	KF977848	KF977852	KF977854	KF977850	Wang et al., 2015a
*P. yunnanensis*	YHHPY1006	KF977849	KF977849	KF977849	KF977853	KF977855	KF977851	Wang et al., 2015a
*P*. sp.	BCC 2637	KF049663	KF049619	KF049637	-	KF049675	KF049693	Kepler et al., 2013
*P*. sp.	JB07 08 16 08	KF049662	KF049616	KF049635	KF049652	KF049672	KF049690	Kepler et al., 2013
*P*. sp.	JB07 08 17 07b	-	KF049617	-	KF049653	KF049673	KF049691	Kepler et al., 2013
*P*. sp.	NBRC 109987	-	-	AB925983	-	-	-	Wang et al., 2021
*P*. sp.	NBRC 109988	-	-	AB925984	-	-	-	Wang et al., 2021
*P*. sp.	NBRC 109990	-	-	AB925968	-	-	-	Wang et al., 2021
*P*. sp.	NBRC 110224	-	-	AB925969	-	-	-	Wang et al., 2021
*P*. sp.	GIMCC 3 570	-	JX006097	JX006098	JX006101	-	JX006100	Wang et al., 2021
*Perennicordyceps cuboidea*	NBRC 101740	JN943321	JN941734	JN941407	JN992468	AB968564	AB968603	Schoch et al., 2012
*Pe. cuboidea*	NBRC 10383	JN943319	JN941735	JN941406	JN992469	AB968563	AB968602	Kepler et al., 2013

Newly generated sequences are indicated in bold. “-” means no data available in GenBank. ARSEF, USDA-ARS Collection of Entomopathogenic Fungal Cultures, Ithaca; BCC, BIOTEC Culture Collection, Khlong Luang, Thailand; CBS, Westerdijk Fungal Biodiversity Institute, Utrecht, Netherlands; CGMCC, China General Microbiological Culture Collection Center, Beijing, China; EFCC, Entomopathogenic Fungal Culture Collection, Chuncheon, Korea; GACP, Herbarium of Guizhou University, China; GZCC, Guizhou Culture Collection, Guizhou Academy of Agricultural Sciences, Guiyang, China; KUMCC, Culture collection of Kunming Institute of Botany, Kunming, China; MFLU, Mae Fah Luang University, Thailand; NBRC, Culture Collection Division Biological Resource Center (NBRC) National Institute of Technology and Evaluation.

### Phylogenetic analyses

Using SeqMan, all newly generated sequences were assembled (Clewley, 1995). The reference taxa for phylogenetic analyses were obtained based on the BLAST search results (https://blast.ncbi.nlm.nih.gov/Blast.cgi) against the non-redundant protein sequence database (NRDB) using default parameters and previously published datasets (Table 1). Individual sequences were aligned using MAFFT v.7 (https://mafft.cbrc.jp/alignment/server/) and trimmed with Trimal v 1.4 (Capella-Gutiérrez et al., 2009; Katoh and Standley, 2013). Alignment was manually adjusted using BioEdit where needed (Hall, 1999). Maximum likelihood (ML) and Bayesian inference (BI) were used to infer phylogenies from a combined six-genetic marker dataset. Outgroup taxa were chosen as *Perennicordyceps cuboidea* (NBRC 101740) and *Pe. cuboidea* (NBRC 103836) (Schoch et al., 2012).

The ML phylogeny was inferred using IQ-TREE 2 with partitioned models and 1,000 exhaustive bootstrap replications (Minh et al., 2020). The model of evolution for each locus was chosen by the built-in ModelFinder tool (Kalyaanamoorthy et al., 2017). The BI analysis was conducted using MCMC sampling and MrBayes version 3.1.2 (Ronquist et al., 2012). The sampling was performed with six simultaneous Markov chains for 1,850,000 generations based on the standard deviation of split frequencies being < 0.01, with trees being sampled every 1,000 generations. The initial 25% of trees were considered as the burn-in phase and were discarded. The posterior probability (PP) was calculated using the remaining trees (Dissanayake et al., 2020). FigTree v.1.4.0 (http://tree.bio.ed.ac.uk/software/figtree/) was used to visualize the ML tree. Based on the guidelines provided by Chethana et al. (2021), Jayawardena et al. (2021), and Maharachchikumbura et al. (2021), novel species descriptions were created.

## Results

### Phylogenetic analyses

**Figure 1:**
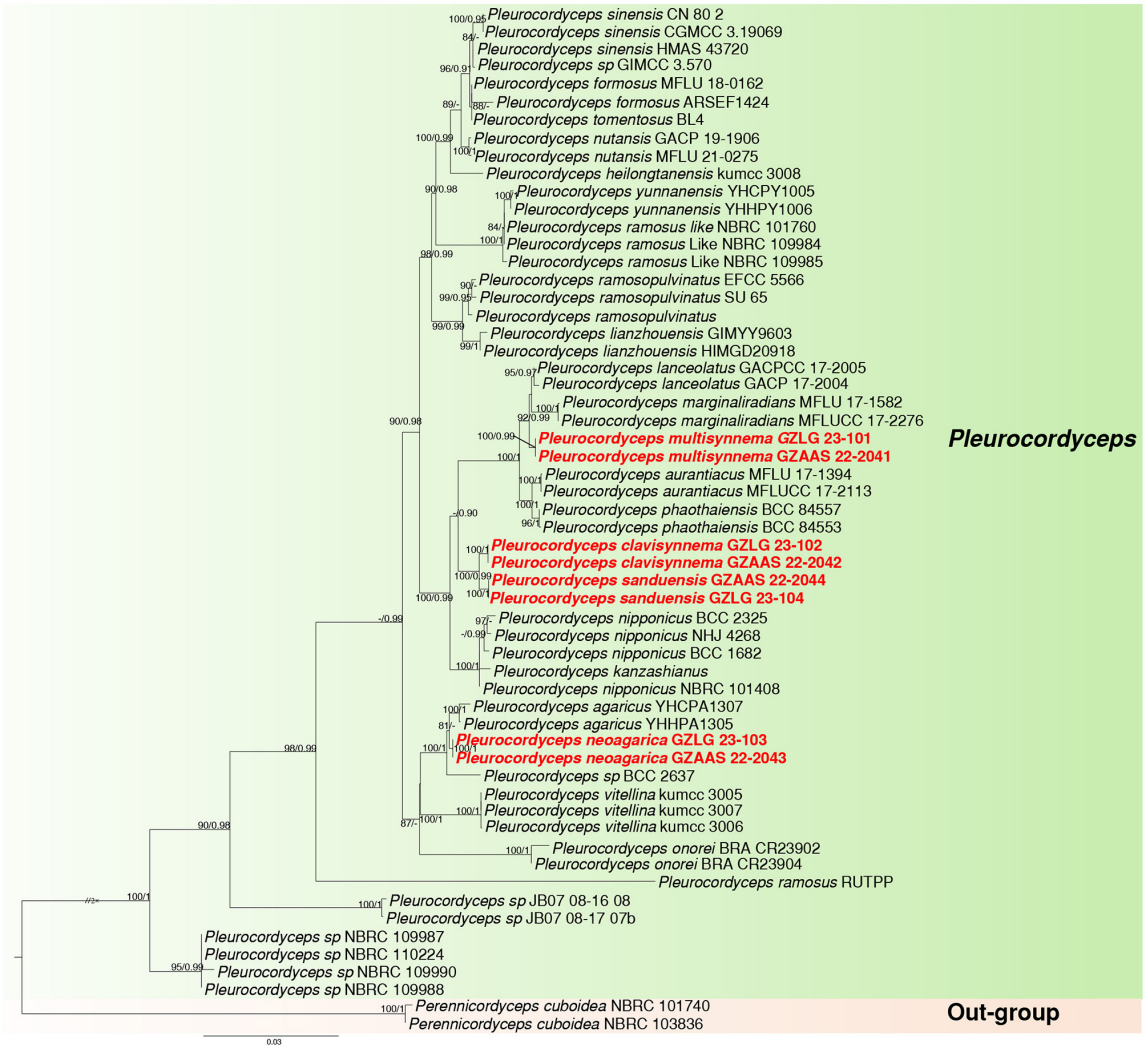
Maximum likelihood phylogenetic tree of 58 taxa and 4,791 sites combining LSU, SSU, ITS, *tef-1*α, *rpb1*, and *rpb2* sequence data. MLBS higher than 75% and PP >0.90 are denoted near the nodes as MLBS/PP, and the newly generated sequences are in red bold font. The genus clade *Pleurocordyceps* is highlighted in green, while the outgroup is marked with a light orange background.

Sequences from 58 taxa representing 24 species of the family Polycephalomycetaceae were obtained from GenBank. The alignment contained 4,791 characteristics, representing 58 taxa. LSU: 847 bp, ITS: 531 bp, SSU: 943 bp, *tef-1*α: 844 bp, *rpb1*: 680 bp, and *rpb2*: 946 bp sequence data, including gaps, were combined in the final alignment. Outgroup taxa included *Perennicordyceps cuboidea* (NBRC 101740) and *Perennicordyceps cuboidea* (NBRC 103836). The topologies of ML and BI analyses were nearly congruent. Figure 1 displays that the maximum likelihood bootstrap (MLBS) is higher than 75%. The collections were determined as four new species, namely, *Pleurocordyceps clavisynnema, P. multisynnema, P. neoagarica*, and *P. sanduensis*. The phylogenetic placement of the new species is described in detail in the notes section below.

### Taxonomy

**Figure 2:**
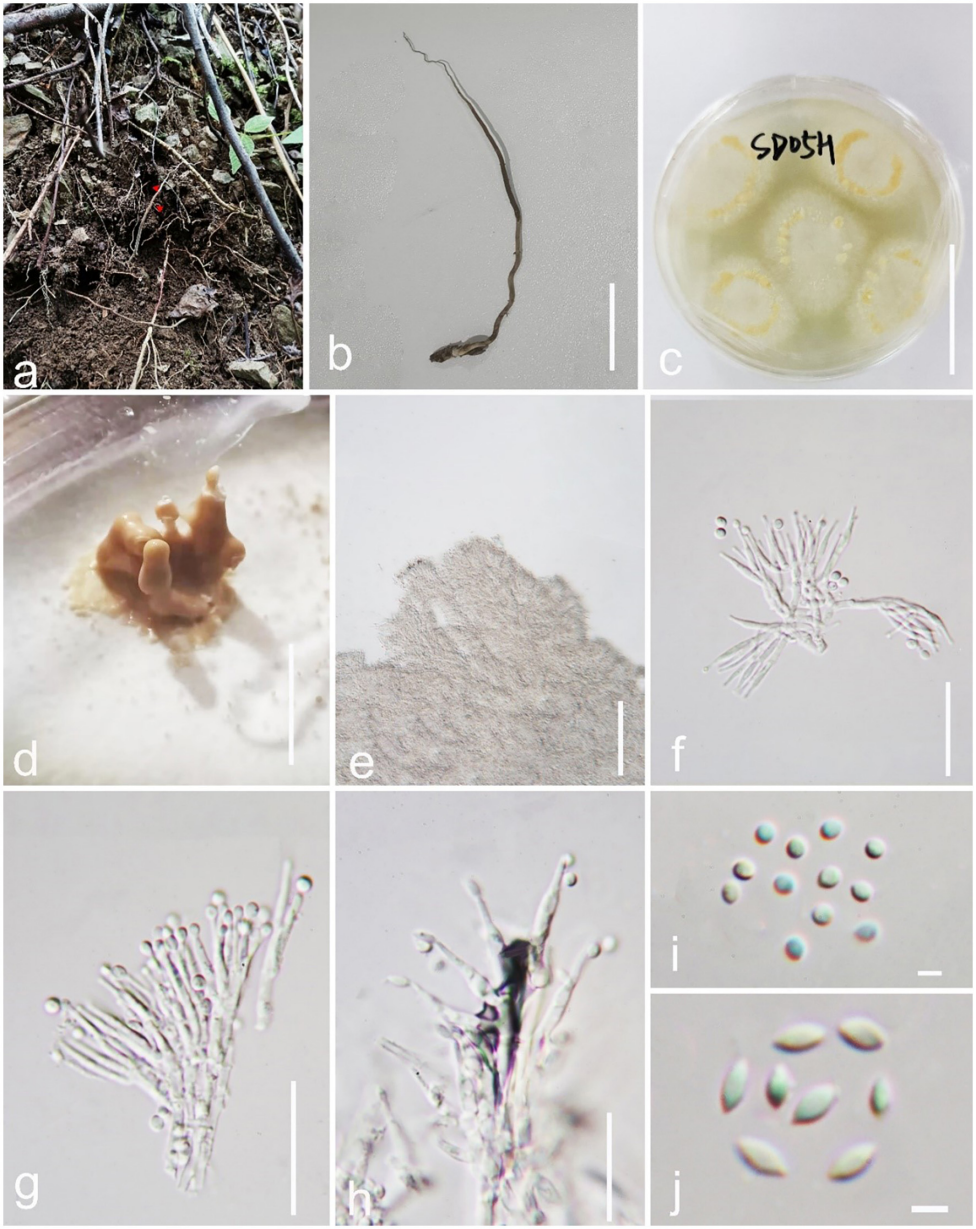
*Pleurocordyceps clavisynnema* (GZLG 23-102, Holotype). **(a, b)** Host: *Ophiocordyceps neogryllotalpae* **(c)** Upper side of the colony. **(d)** Synnemata on the culture. **(e)** Conidiophores. **(f, g)** α-phialides. **(h)** β-phialides. **(i)** α-conidia. **(J)** β-conidia. Scale bars: **(b, c)** 3 cm, **(d)** 0.5 cm, **(e)** 100 μm, **(f–h)** 20 μm, **(i, j)** 3 μm.

*Pleurocordyceps clavisynnema* Y. P. Xiao and Y. Yang sp. nov (Figure 2).

*Index Fungorum number*: IF900449; Faceoffungi number: FoF 14158
*Etymology*: Name referring to clavate synnemata.
*Holotype*: GZLG 23-102

*Parasitism* on *Ophiocordyceps neogryllotalpae* (*Ophiocordycipitaceae, Hypocreales*). Sexual morph: Not observed. Asexual morph: Hyphomycetous. Culture characteristics: *Colonies* on PDA fast-growing, derived from tissue isolation, reaching 3 cm wide in 2 weeks at 25°C, white, and obverse brown. *Synnemata* emerging after 20 days, clavate or with a mucronate apex, solitary, unbranched, and 2–5 mm long. *Fertile head* 0.6–2.3 mm wide, yellowish to yellow, emerging on the middle part of the synnemata or on the top, with conidial masses on the surface. *Conidial masses* brown, slimy. *Conidiophore* 21–39 μm long ($\bar{x}$ = 20 μm, *n* = 40), 2–6 phialides in one. *Phialides* has two types α*-phialides* 8.3–14.5 × 0.9–1.7 μm ($\bar{x}$ = 11.4 × 1.3 μm, *n* = 40) smooth, hyaline, solitary. β*-phialides* 12.3–21.6 × 0.8–1.8 μm ($\bar{x}$ =16.95 × 1.3 μm, *n* = 40), smooth, hyaline, solitary. α*-conidia* 1.7–2.6 μm (= 2.15 μm, *n* = 50) wide, globose, 1-celled, smooth-walled; β*-conidia* 3.1–4.1 × 1.6–2.2 μm ($\bar{x}$ = 3.6 × 1.9 μm, *n* = 50), hyaline, fusiform, 1-celled, smooth.

*Material examined*: China, Guizhou Province, Qiannan Buyi and Miao Autonomous Prefecture, Sandu Shui Autonomous County. Parasitic on *Ophiocordyceps neogryllotalpae* (*Ophiocordycipitaceae, Hypocreales*), in the soil, 10 April 2022, Yu Yang, SD05H (GZLG 23-102, holotype; ex-type living culture, GZCC 22-2042).

*Notes*: *Pleurocordyceps sanduensis* is the closest match to our new sample of *P. clavisynnema*. This is also confirmed by phylogenetic analyses, whereby the two are sister taxa with maximum statistical support (100% ML/1.00 PP; Figure 1). Base pair differences between *P. clavisynnema* and *P. sanduensis* are 23/824 in *tef-1*α, 8/1130 in SSU, 2/678 in *rpb1*, and 3/1050 in *rpb2*. Morphologically, *P. clavisynnema* differs from *P. sanduensis* by having longer synnemata, larger conidiophore, smaller phialides, and shorter conidia. Hence, this study introduces *Pleurocordyceps clavisynnema* as a new species based on morphological and phylogenetic analyses.

**Figure 3:**
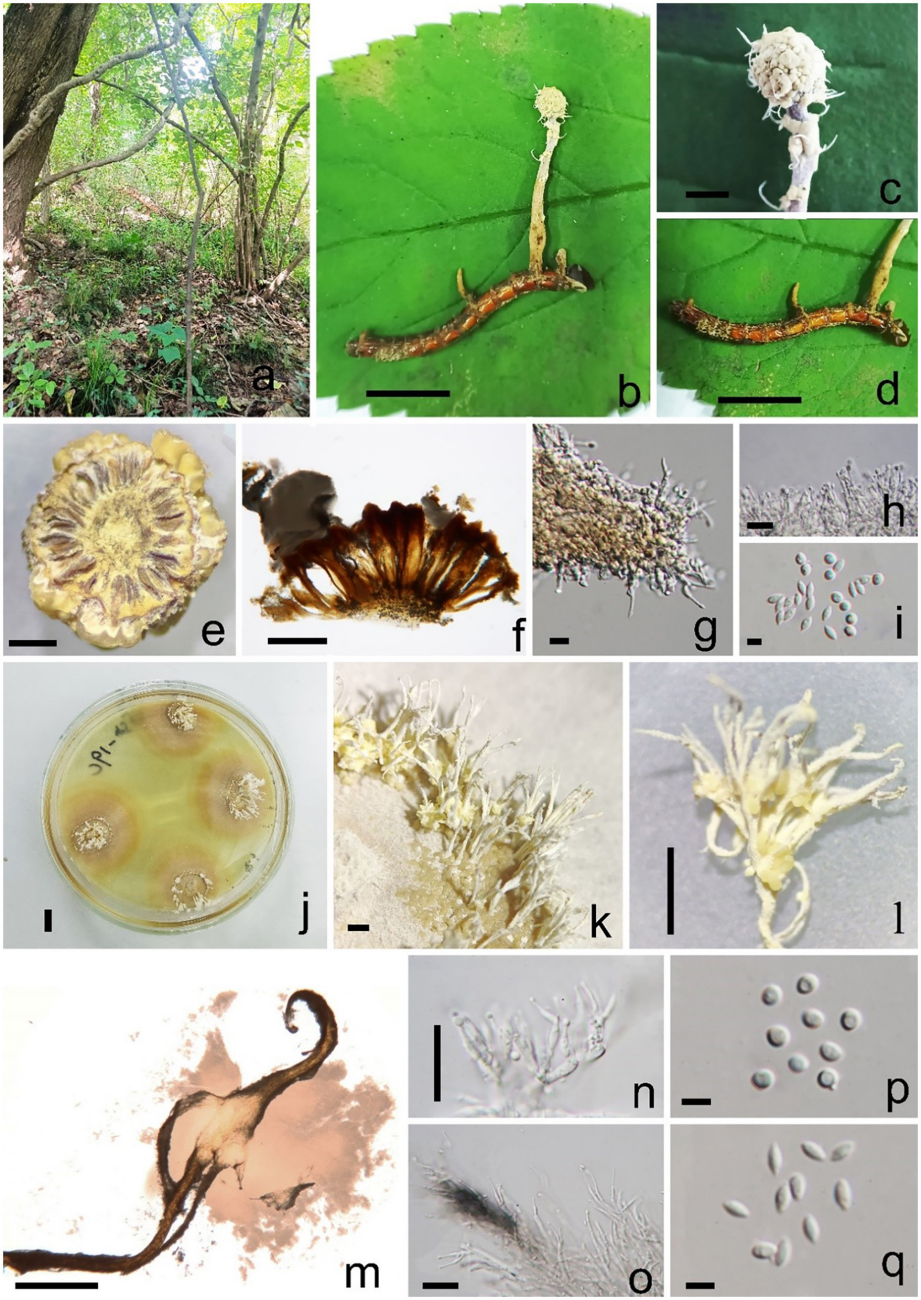
*Pleurocordyceps multisynnema* (GZLG 23-101, holotype) **(a)** Habitat. **(b)** Overview of *Pleurocordyceps multisynnema*. **(c)** Synnemata on the host. **(d)** Host of *Paraisaria* sp. **(e, f)** Section of host. **(g)** β-phialides. **(h)** α-phialides. **(i)** α-conidia and β-conidia. **(j)** Culture from above on PDA medium. **(k–m)** Synnemata on the culture. **(n)** α-phialides. **(o)** β-phialides. **(p)** α-conidia. **(q)** β-conidia. Scale bars: **(b, d, j)** 1 cm, **(c)** 0.2 cm, **(e)** 300 μm, **(f)** 200 μm, **(g, h)** 10 μm, **(i, p, q)** 3 μm, **(k, l)** 0.5 cm, **(m)** 500 μm, **(n, o)** 20 μm.

*Pleurocordyceps multisynnema* Y. Yang and Y. P. Xiao sp. nov (Figure 3).

*Index Fungorum number*: IF900451; *Faceoffungi number*: FoF 14160
*Etymology*: Name referring to the multiple synnemata of the host and culture.
*Holotype:* GZLG 23-101

Sexual morph: absent. Asexual morph: *Synnemata* generating from the fertile head of the host, single, light yellow, cylindrical, without a fertile head, stipitate, usually unbranched. *Conidial mass* yellowish, covered the surfaces of the host. α*-phialides* 9–15 × 1.1–2.2 μm ($\bar{x}$ = 12 × 1.65 μm, *n* = 40), solitary, narrow lanceolate, from the synnema. β*-phialides* 19.8–25.9 × 1.7–2.6 μm ($\bar{x}$ = 22.85 × 2.15 μm, *n* = 40), directly from hyphae, solitary, narrow lanceolate, suddenly tapering from the bottom to the apex. *Conidia* one-celled, hyaline, smooth, two types. α*-conidia* 2.1–2.5 μm ($\bar{x}$ = 2.3 μm, *n* = 50), spherical, one-celled, smooth. β*-conidia* 2.9–3.8 × 1.3–2.2 μm ($\bar{x}$ = 3.7 × 1.9 μm, *n* = 50), fusiform, one-celled, smooth.

*Colonies* on PDA medium slow-growing, isolated from the tissue of synnemata, circular, attaining 3 cm in 35 days at 25°C, dry yellow. *Synnemata* arising the margin of the colony after 30 days, without a fertile head, solitary or two- or three-branched, 2–6 × 0.9–1.8 mm ($\bar{x}$ = 4 × 1.35 mm, *n* = 30), with several radiating ring-like distributions. *Conidial masses* pale yellow to yellow, covered the surface of the colony or generated from the middle part of the synnemata with hyaline to white yellow slime. *Conidiophore* 2–4 phialides in one. α*-phialides* 9–13.4 × 0.9–1.3 μm ($\bar{x}$ = 11.2 × 1.1 μm, *n* = 40) unbranched, hyaline, smooth. β*-phialides* 12.8–20.9 × 1.9–2.8 μm ($\bar{x}$ = 16.85 × 2.35 μm, *n* = 40), solitary, generating from hyphae laterally, hyaline, smooth. α*-conidia* 1.7–2.5 μm wide ($\bar{x}$ = 2.1 μm, *n* = 50), globose, one-celled, smooth-walled; β*-conidia* 2.6–3.5 × 1.3–2.2 μm ($\bar{x}$ = 3.05 × 1.75 μm, *n* = 50) hyaline, 1-celled, fusiform, smooth-walled.

*Material examined*: China, Anhui Province, Chuzhou City, parasitic on *Paraisaria* sp., on leaf litter, 25 August 2021, Yu Yang, HFS19a (GZLG 23-101, holotype; ex-type living culture, GZCC 22-2041).

*Notes: Pleurocordyceps multisynnema* has a high support value (100% ML/1 PP) and is sister to *P. lanceolatus* and *P. marginaliradians* in the phylogenetic tree (Figure 1). Comparing the ITS, LSU, SSU, *tef-1*α, *rpb1*, and *rpb2* sequences of *P. multisynnema* and *P. lanceolatus* revealed 97.89% (12 bp differences), 99.28% (5 bp differences), 99.27% (6 bp differences), 99.77% (2 bp differences), 98.38% (11 bp differences), and 98.97% (10 bp differences) sequence similarities, respectively. *Pleurocordyceps multisynnema* differs from *P. lanceolatus* in that it is parasitic on *Paraisaria* species and produces conidia that range from coiled to thread-like but lack fertile heads (Xiao et al., 2023). *Pleurocordyceps multisynnema* differs from *P. marginaliradians* in distinct hosts (*Paraisaria* sp. vs. *Cossidae* larva), shorter phialides, and conidia (Xiao et al., 2018). As a result, *Pleurocordyceps multisynnema* is described as a new species of *Pleurocordyceps*.

**Figure 4:**
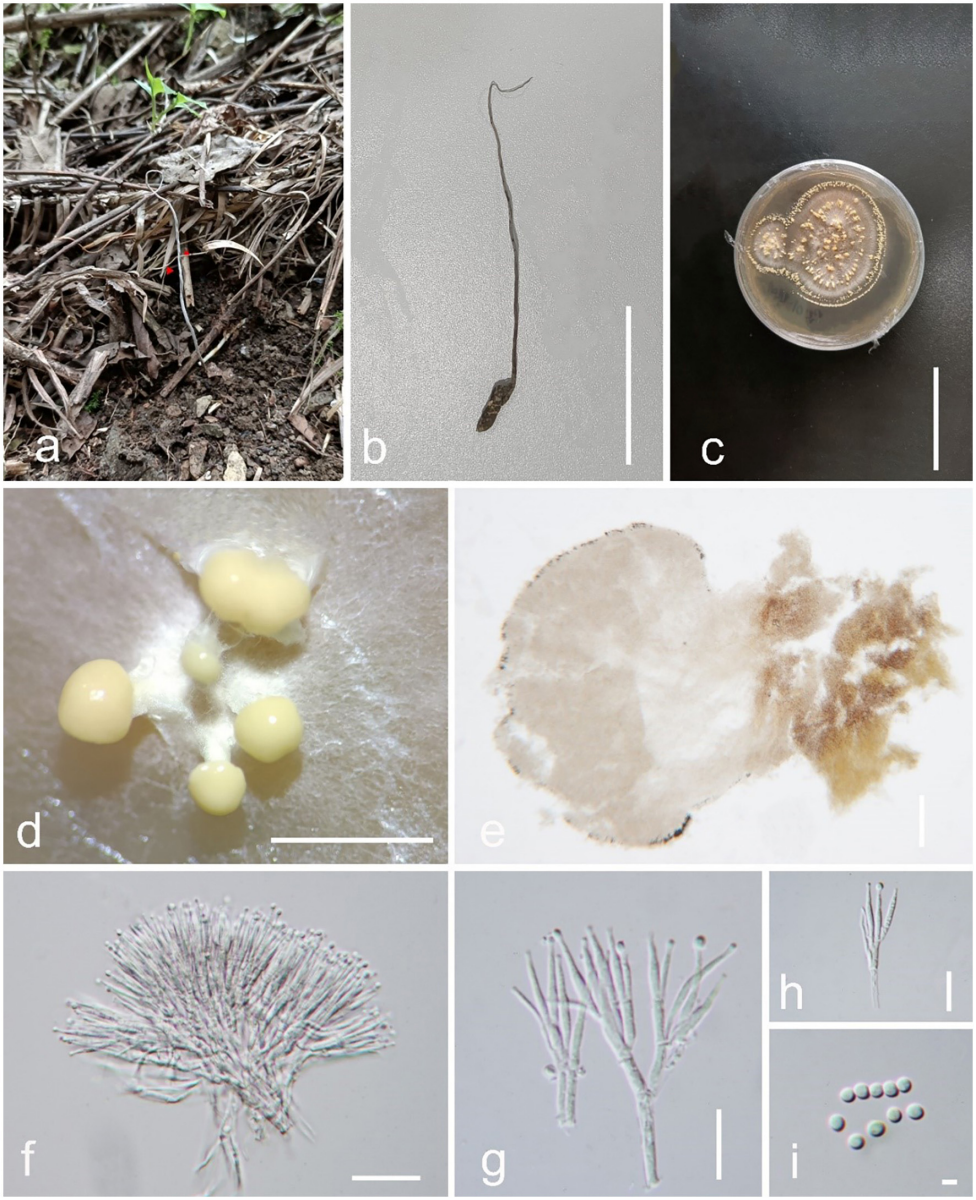
*Pleurocordyceps neoagarica* (GZLG 23-103, Holotype). **(a, b)** Host: *Ophiocordyceps neogryllotalpae* **(c)** Upper side of the colony. **(d, e)** Synnemata on the culture. **(f)** Conidiophores. **(g, h)** Phialides. **(i)** Conidia. Scale bars: **(b, c)** 5 cm, **(d)** 3 mm, **(e)** 200 μm, **(f)** 20 μm, **(g, h)** 10 μm, **(i)** 2 μm.

*Pleurocordyceps neoagarica* Y. Yang and Y. P. Xiao sp. nov (Figure 4).

*Index Fungorum number*: IF900450; *Faceoffungi number*: FoF 14159
*Etymology*: Name referring to the similar species, *Pleurocordyceps agarica*.
*Holotype:* GZLG 23-103

*Parasitism* on *Ophiocordyceps neogryllotalpae* (Ophiocordycipitaceae, Hypocreales). Sexual morph: Not observed. Asexual morph**:** Hyphomycetous. Culture characteristics: *Colonies* on PDA quickly grown, isolated from the tissue, reaching 5 cm wide in 25 days at 25°C, white, reverse brown. *Synnemata* appearing after 15 days, 0.5–3 mm long, solitary, non-branched, displaying several ring-like distributions. *Fertile head* 1.2–2.3 mm wide, globose, pale yellow, producing from the top of the synnemata. *Conidial masses* covered the surface of synnemata or the top of synnemata, white yellow, slimy. *Conidiophore* 42–63 μm long ($\bar{x}$ = 52.5 μm, *n* = 40), 2–4 phialides in one. *Phialides* 11.6–17.4 × 1.1–1.9 μm ($\bar{x}$ = 14.5 × 1.5 μm, *n* = 50), one type, narrowly slim lanceolate, cylindrical at the base, 6–13 μm long, tapered into a long neck, 1.2–3.1 μm long, hyaline, smooth. *Conidia* 2.1–2.9 μm ($\bar{x}$ = 2.5 μm, *n* = 50), arising from the apex of phialides, globose, 1-celled, hyaline.

*Material examined*: China, Guizhou Province, Qiannan Buyi and Miao Autonomous Prefecture, Sandu Shui Autonomous County. Parasitic on *Ophiocordyceps neogryllotalpae* (Ophiocordycipitaceae, Hypocreales), in the soil, 10 April 2022, Yu Yang, SD10H (GZLG 23-103, holotype; ex-type living culture, GZCC 22-2043).

*Notes*: *Pleurocordyceps neoagarica* (Host: *Ophiocordyceps neogryllotalpae*) differs from *P. agarica* (Host: *Ophiocordyceps barnesii*) morphologically due to its distinct host, longer synnemata and conidiophore, and shorter phialides (Wang et al., 2015b). *P. neoagarica* produces only one type of phialides and conidia, whereas *P. agarica* produces two. In the phylogenetic tree, the new collections (GZLG 23-103) shared a sister relationship with *Pleurocordyceps agarica* (Figure 1). The type of strain of *P. neoagarica* differs from *P. agarica* by 4 bp in ITS, 7 bp in SSU, 4 bp in *rpb1*, and 14 bp in *rpb2* (Wang et al., 2015b). Given the significant morphological differences between these two taxa and their distinct phylogenetic placement, we conclude that they are separate species.

**Figure 5:**
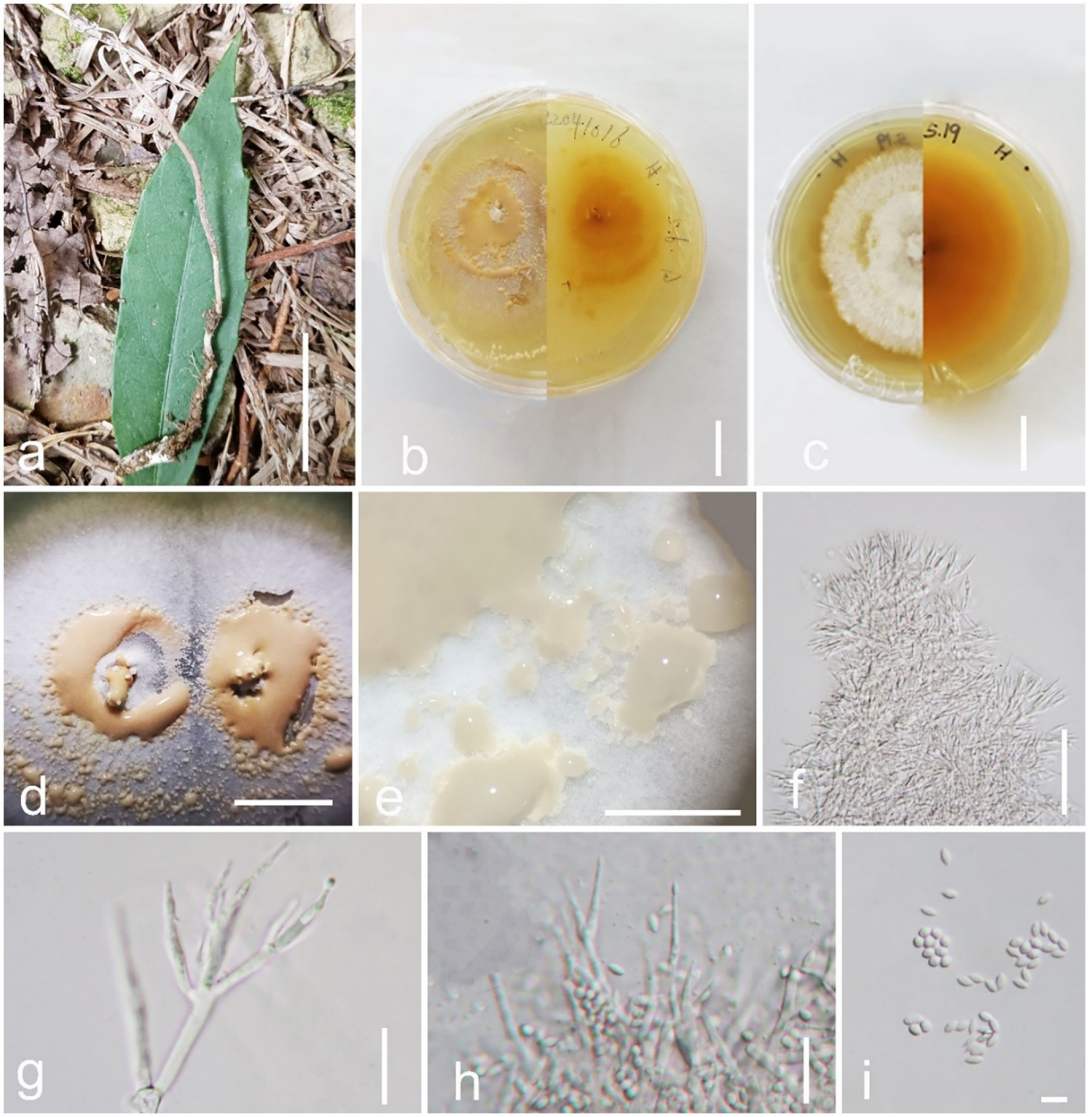
*Pleurocordyceps sanduensis* (GZLG 23-104, Holotype). **(a)** Host: *Ophiocordyceps neogryllotalpae* **(b, c)** Upper and back side of the colony. **(d, e)** Conidial masses on the culture. **(f)** Conidiophores. **(g)** α-phialides. **(h)** β-phialides **(I)** α-conidia and β-conidia. Scale bars: **(a)** 5 cm; **(b**–**d)** 1 cm, **(e)** 0.5 cm, **(f)** 50 μm, **(g)** = 10 μm, **(h)** 20 μm, **(i)** 5 μm.

*Pleurocordyceps sanduensis* Y. P. Xiao and Y. Yang sp. nov (Figure 5).

*Index Fungorum number*: IF900447; *Faceoffungi number*: FoF 14157
*Etymology*: Name referring to the locality Sandu County.
*Holotype:* GZLG 23-104

*Parasite* on *Ophiocordyceps neogryllotalpae* (Ophiocordycipitaceae, Hypocreales). Sexual morph: Not observed. Asexual morph**:** Hyphomycetous. Culture characteristics: *Colonies* on PDA fast-growing, obtained from tissue, reaching 5 cm wide in 20 days at 25°C, white, reverse yellow to brown, presenting multiple radiating ring-like distributions. *Synnemata* emerging after 25 days, solitary, unbranched, 0.1–0.5 mm long, distribution at the edge, with small or without a fertile head. *Conidial masses* covered the surface of the colony, pale yellow when young, later change to brown color, slime. *Conidiophore* 12–23 μm long ($\bar{x}$ = 17.5 μm, *n* = 30), multiple phialides in one. *Phialides* exist in α*-phialides* and β*-phialides*. α*-phialides* 9.5–18.7 × 0.8–2.1 μm ($\bar{x}$ = 14.1 × 1.45 μm, *n* = 40), smooth, hyaline, solitary. β*-phialides* 19–33.4 × 0.9–1.8 μm ($\bar{x}$ = 26.2 × 1.35 μm, *n* = 40), smooth, hyaline, solitary. α*-conidia* 2.1–3.1 μm ($\bar{x}$ = 2.6 μm, *n* = 50) wide, globose, unicellular, smooth-walled; β*-conidia* 3.3–5.5 × 1.5–2.1 μm ($\bar{x}$ = 4.4 × 1.8 μm, *n* = 50) fusiform, unicellular, hyaline, smooth-walled.

*Material examined*: China, Guizhou Province, Qiannan Buyi and Miao Autonomous Prefecture, Sandu Shui Autonomous County. Parasitic on *Ophiocordyceps neogryllotalpae* associated with the larva of *Gryllotalpa* species, in soil, collected on 10 April 2022, Xingcan Peng, SD16 (GZLG 23-104, holotype; ex-type living culture, GZCC 22-2044).

*Notes*: *Pleurocordyceps sanduensis* (holotype: GZLG 23-104) is sister to *P. clavisynnema* (holotype: GZLG 23-102) with maximum statistical support (100% ML/1.00 PP) (Figure 1). *Pleurocordyceps sanduensis* is isolated from the same host as *P. clavisynnema*. However, the two are distinct in terms of both morphology and phylogeny. Base pair differences between *P. clavisynnema* and *P. sanduensis* are 23/824 in *tef-1*α, 8/1130 in SSU, 2/678 in *rpb1*, and 3/1050 in *rpb2*. Morphologically, *Pleurocordyceps sanduensis* differs from *P. clavisynnema* in shorter synnemata, smaller conidiophore, larger phialides, and longer conidia. Hence, this study introduces *P. clavisynnema* as a new species based on morphological and phylogenetic analyses.

## Discussion

Herein, we describe four new species of *Pleurocordyceps* (*P. clavisynnema, P. multisynnema, P. neoagarica*, and *P*. *sanduensis*) using a combination of morphology and phylogeny. The newly established species group distinctly form independent clades in the phylogenetic tree (Figure 1). Morphologically, three of the new species (*P. clavisynnema, P. multisynnema*, and *P. sanduensis*) are similar to *P. aurantiacus, P. agarica, P. heilongtanensis, P. lanceolatus, P. marginaliradians, P. nutansis, P. sinensis, P. vitellina*, and *P. yunnanensis* in that they have two types of phialides and conidia. However, the hosts on which *P. clavisynnema, P. multisynnema*, and *P. sanduensis* parasitize differ from those of other species of *Pleurocordyceps* (Wang et al., 2012, 2015a,b; Xiao et al., 2018, 2023). Meanwhile, *P. neoagarica* is similar to *P. lianzhouensis* and *P. parvicapitata* in that it has one type of phialides and conidia (Wang et al., 2014; Xiao et al., 2023). However, *P. neoagarica* differs from *P. lianzhouensis* and *P. parvicapitata* as it parasitizes different hosts and produces longer phialides and smaller conidia (Wang et al., 2014; Xiao et al., 2023).

The discovery of the new species of *Pleurocordyceps* adds to the diversity of the genus and the associated family. Several *Pleurocordyceps* taxa have been found in China, indicating a high diversity of these organisms in the country. *Pleurocordyceps* species display variable host specialization (Wang et al., 2012; Xiao et al., 2023). A few are host-specific. Herein, *Pleurocordyceps clavisynnema, P. neoagarica*, and *P. sanduensis* were isolated from the same host, *Ophiocordyceps neogryllotalpae*. This is similar to the previous results, whereby *P. nutansis* and *P. yunnanensis* are parasitic on the same fungus, *Ophiocordyceps nutans* (Wang et al., 2015a; Xiao et al., 2023). Most *Pleurocordyceps* taxa are not host-specific, and multiple species have been documented in the same host (Bischoff et al., 2003; Wang et al., 2012, 2015a,b; Matočec et al., 2014; Crous et al., 2017; Xiao et al., 2018). Members of the genus parasitize insects and fungi, several species of which have broad geographic distributions possibly reflecting the diversity of *Pleurocordyceps* habitats. Future studies should focus on collecting additional *Polycephalomycetaceae* taxa to not only uncover the full extent of diversity of this family but also understand their distribution in relation to their hosts.

## Data availability statement

The data presented in the study are deposited in the Guizhou Institute of Technology herbarium, accession number GZLG 23-102, GZCC 22-2042, GZLG 23-101, GZCC 22-2041, GZLG 23-103, GZCC 22-2043, GZLG 23-104, and GZCC 22-2044.

## Author contributions

Y-PX: Writing – original draft. YY: Writing – original draft. RJ: Writing – review & editing. EG: Writing – review & editing. X-CP: Formal analysis, Writing – review & editing. Z-LL: Writing – review & editing. Y-ZL: Writing – review & editing.
